# Potential Pitfalls of Using Fractional Anisotropy, Axial Diffusivity, and Radial Diffusivity as Biomarkers of Cerebral White Matter Microstructure

**DOI:** 10.3389/fnins.2021.799576

**Published:** 2022-01-14

**Authors:** Chase R. Figley, Md Nasir Uddin, Kaihim Wong, Jennifer Kornelsen, Josep Puig, Teresa D. Figley

**Affiliations:** ^1^Department of Radiology, University of Manitoba, Winnipeg, MB, Canada; ^2^Division of Diagnostic Imaging, Health Sciences Centre, Winnipeg, MB, Canada; ^3^Neuroscience Research Program, Kleysen Institute for Advanced Medicine, Winnipeg, MB, Canada; ^4^Department of Physiology & Pathophysiology, University of Manitoba, Winnipeg, MB, Canada; ^5^Department of Neurology, University of Rochester, Rochester, NY, United States; ^6^Girona Biomedical Research Institute (IDIBGI), Hospital Universitari de Girona Dr. Josep Trueta, Girona, Spain

**Keywords:** axial diffusivity, crossing fibers, diffusion MRI, fractional anisotropy, radial diffusivity, white matter

## Abstract

Fractional anisotropy (FA), axial diffusivity (AD), and radial diffusivity (RD) are commonly used as MRI biomarkers of white matter microstructure in diffusion MRI studies of neurodevelopment, brain aging, and neurologic injury/disease. Some of the more frequent practices include performing voxel-wise or region-based analyses of these measures to cross-sectionally compare individuals or groups, longitudinally assess individuals or groups, and/or correlate with demographic, behavioral or clinical variables. However, it is now widely recognized that the majority of cerebral white matter voxels contain multiple fiber populations with different trajectories, which renders these metrics highly sensitive to the relative volume fractions of the various fiber populations, the microstructural integrity of each constituent fiber population, and the interaction between these factors. Many diffusion imaging experts are aware of these limitations and now generally avoid using FA, AD or RD (at least in isolation) to draw strong reverse inferences about white matter microstructure, but based on the continued application and interpretation of these metrics in the broader biomedical/neuroscience literature, it appears that this has perhaps not yet become common knowledge among diffusion imaging end-users. Therefore, this paper will briefly discuss the complex biophysical underpinnings of these measures in the context of crossing fibers, provide some intuitive “thought experiments” to highlight how conventional interpretations can lead to incorrect conclusions, and suggest that future studies refrain from using (over-interpreting) FA, AD, and RD values as standalone biomarkers of cerebral white matter microstructure.

## Brief Background

The popularity of diffusion MRI (dMRI) has increased dramatically over the past couple of decades, and it is now commonly used for a wide range of clinical and research applications (Lerner et al., 2014; Assaf et al., 2019). Indeed, it is quite remarkable how much, and even how many different types of information can be gleaned from the endogenous diffusion characteristics of water molecules within our brains. For example, dMRI data has been used to: (1) derive several different quantitative measures [e.g., fractional anisotropy, axial diffusivity, radial diffusivity, mean diffusivity (Beaulieu, 2002; Alexander et al., 2019); axial kurtosis, radial kurtosis, mean kurtosis, maximum directional kurtosis, axonal water fraction (Fieremans et al., 2011; Henriques et al., 2021); neurite orientation dispersion, neurite density index, isotropic volume fraction (Zhang et al., 2012; Faiyaz et al., 2021); etc.] that reflect slightly different aspects of tissue microstructure, (2) non-invasively map the brain’s white matter pathways using deterministic (Mori et al., 1999) and/or probabilistic (Behrens et al., 2003) tractography approaches (Maier-Hein et al., 2017; Jeurissen et al., 2019), and (3) indirectly measure brain function (Le Bihan et al., 2006b; Le Bihan, 2007; Abe et al., 2017). It is therefore not surprising that researchers are leveraging these techniques to study diffusion changes associated with neurodevelopment (Lebel et al., 2019), brain aging (Beck et al., 2021), traumatic brain injury (Hutchinson et al., 2018), and variety of neurodegenerative disorders (Goveas et al., 2015) – in many cases to correlate one or more quantitative dMRI metrics in various brain regions with developmental, demographic, clinical, and/or cognitive measures.

With that being said, along with the power and flexibility of dMRI comes a number of complications, caveats and limitations – and indeed, some excellent articles have been written about common pitfalls associated with the acquisition, analysis and interpretation of dMRI data (Le Bihan et al., 2006a; Jones and Cercignani, 2010; Jones et al., 2013). These papers are excellent resources, and expertly explain potential problems and mitigation strategies associated with common dMRI methods, and will therefore not be recapitulated here. Additionally, there have been several excellent in-depth reviews published on the biophysical interpretations of diffusion MRI signals (Beaulieu, 2002; Jelescu and Budde, 2017; Jelescu et al., 2020). Therefore, the limited focus of the current manuscript is to briefly highlight and discuss (in non-technical language) the potential “pitfall” of over-interpreting fractional anisotropy (FA) – and for similar reasons, axial diffusivity (AD) and radial diffusivity (RD) – in the presence of white matter crossing fibers. Problems associated with complex white matter fiber geometries are well established among dMRI fiber tracking (a.k.a., tractography) experts, who have developed various data acquisition and analysis methods to resolve these issues (Maier-Hein et al., 2017; Jeurissen et al., 2019).

However, diffusion tensor imaging (DTI) is one of the most well established and widely used dMRI approaches, with ‘‘plug-and-play’’ MRI pulse-sequences and practically ‘‘push-button’’ analysis packages, which makes it accessible to a wide variety of end-users with varying degrees of technical knowledge. We therefore aim to highlight how abundant crossing fibers are in cerebral white matter, and explain in non-technical language how this creates inherent problems for drawing reverse inferences about underlying tissues based on FA, AD, and RD measures. We will then walk readers through two intuitive ‘‘thought experiments’’ to illustrate how conventional interpretations of FA, AD, and RD measures can lead to incorrect inferences about the underlying white matter tissues based on conventional interpretations, and conclude by discussing some alternative MRI methods that are likely more robust to white matter fiber crossings. In so doing, we hope to warn DTI practitioners about using FA, AD, and RD measures as quantitative biomarkers of cerebral white matter, and to discourage future studies from using these metrics in isolation to compare cross-sectional ‘‘differences’’ between individuals or groups, longitudinal ‘‘changes’’ within individuals, and/or to correlate with demographic, clinical, or behavioral/neuropsychological data, which have been (and continue to be) commonly reported in the dMRI literature^1^.

## Introducing the Problem (Pitfall)

Fractional anisotropy in particular (as well as AD and RD to a lesser extent) has long been one of the most commonly reported quantitative dMRI metrics. It can be obtained from dMRI scans with even relatively low *b*-values (≥700 s/mm^2^) and a relatively small number of diffusion-encoding directions (≥6 orthogonal directions) (Alexander et al., 2007), is very easy to calculate (based on the common tensor model) (Basser and Pierpaoli, 1996), yields high test-retest and even inter-site/cross-scanner reliability (Vollmar et al., 2010; Luque Laguna et al., 2020), and generally shows high correlations with other quantitative MRI metrics (Uddin et al., 2019).

**FIGURE 1:**
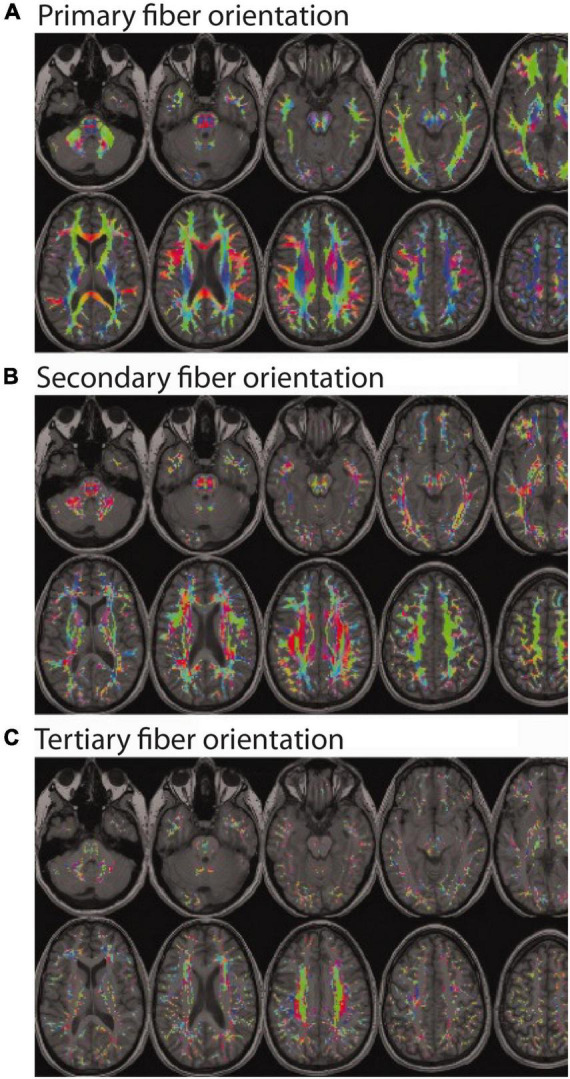
Color encoded maps of white matter fiber directions (red = left/right; green = anterior/posterior; blue = superior/inferior). Colored regions indicate white matter locations with: **(A)** at least one fiber population (and the orientation of the main bundle); **(B)** at least two fiber populations (and the orientation of the secondary bundle); and **(C)** at least three fiber populations (and the orientation of the tertiary bundle). Even a cursory visual comparison of **(A,B)** reveals that most white matter regions contain at least two fiber populations with different orientations (i.e., crossing fibers), and a closer inspection of **(A,C)** reveals a non-trivial number of regions with three or more fiber populations with different orientations. (Note: Figure modified and reproduced with permission from Dr. Ben Jeurissen and John Wiley and Sons Publishers via the Copyright Clearance Center. Original version published in Jeurissen et al., 2013).

Within the framework of DTI, AD is the amount of apparent diffusion along the principal diffusion axis (AD = λ_1_), RD is the average amount of apparent diffusion along the secondary and tertiary diffusion axes (RD = [λ_2_ + λ_3_]/2), and mean diffusivity (MD) is the average amount of apparent diffusion along each of the three diffusion axes (MD = [λ_1_ + λ_2_ + λ_3_]/3). As its name implies, FA is a relative measure of diffusion anisotropy within a given voxel or region (FA = $\sqrt{\frac{1}{2}}\,\frac{\sqrt{{\left(\lambda \,1-\lambda \,2\right)}^{2}+{\left(\lambda \,1-\lambda \,3\right)}^{2}+{\left(\lambda \,2-\lambda \,3\right)}^{2}}}{\sqrt{{\left(\lambda \,1\right)}^{2}+{\left(\lambda \,2\right)}^{2}+{\left(\lambda \,3\right)}^{2}}}$), which indicates the amount of diffusion in the principal direction compared to the orthogonal two directions. The values of FA are therefore unitless and inherently scaled between 0 (i.e., equal amounts of diffusion in all directions) and 1 (i.e., diffusion in only one direction). Empirically, FA values are uniformly low in gray matter, high in most white matter regions, and by contrast (no pun intended) are relatively low in focal white matter lesions. Therefore, until relatively recently, the conventional thinking was that all else being equal, higher FA values generally reflected greater white matter density – except for a few widely recognized exceptions with known crossing fibers [e.g., at the intersections between the corpus callosum and the ascending/descending corona radiata, as well as between the superior longitudinal fasciculus and the corona radiata (Tuch et al., 2003)], where this common interpretation was acknowledged to be problematic. It has long been known that the restriction of water diffusion in white matter depends on multiple factors, including: fiber diameter, fiber density, membrane permeability, myelination, and the directional organization/coherence of these boundaries (Beaulieu, 2002). Indeed, the complex interactions between these factors and the resulting inability to ascribe apparent diffusion changes to any particular cause, is why (Jones et al., 2013) and others have advocated for researchers to exercise caution when interpreting DTI data and avoid drawing conclusions about microstructural or tissue “integrity.” However, because the proportion of cerebral white matter voxels containing complex fiber geometries and/or multiple fiber bundles in different orientations (i.e., crossing fibers, kissing fibers, etc.) is now thought to be at least 33% (Behrens et al., 2007), and is more likely somewhere between 60 and 90% (Jeurissen et al., 2013) – with many regions thought to contain as many as 3 or more intersecting fiber bundles with different trajectories (Figure 1) – this makes the conventional “if some is good, more is better” interpretation of FA values much more problematic than originally thought.

## Demonstrating the Problem (Pitfall)

The inherent nature of this problem, and its level of complexity can perhaps be most easily illustrated using two brief thought experiments.

### Thought Experiment #1: Interpreting Fractional Anisotropy Differences or Changes (Increases and Decreases)

**FIGURE 2:**
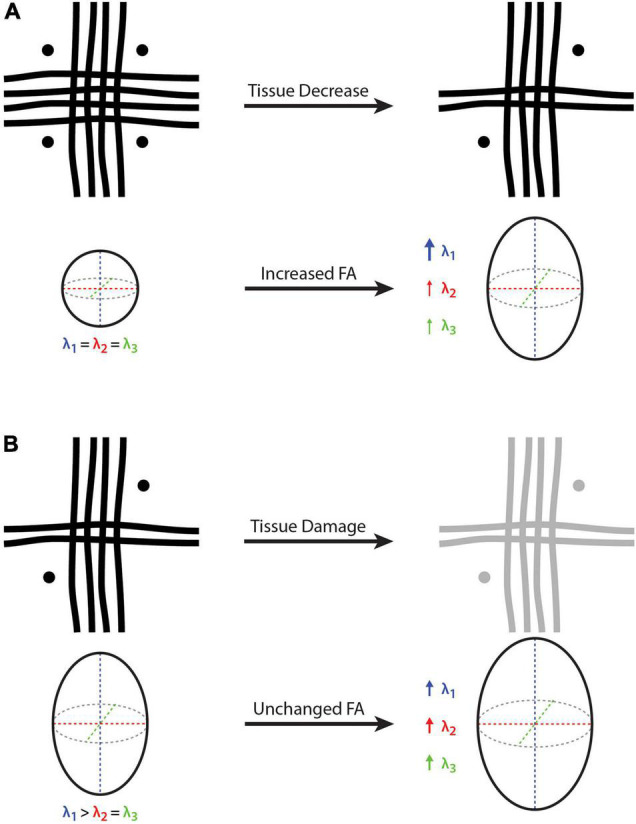
Cartoon depictions of a voxel containing three white matter crossing fibers (i.e., vertical, horizontal, and through-plane fibers) and the corresponding diffusion tensor and fractional anisotropy (FA). **(A)** On the left-hand side, FA = 0 because there is no dominant fiber direction (i.e., same diameter, density and integrity of fibers arranged orthogonally), where water diffusion will be constrained equally in all directions. On the right-hand side, the vertical fibers remain unchanged while the other two fiber bundles (i.e., horizontal and through-plane) are decreased, such that λ_1_ becomes larger than λ_2_ and λ_3_, resulting in an FA increase despite a net white matter fiber reduction. **(B)** On the left-hand side, there is a principle fiber orientation (i.e., in the vertical direction) and a corresponding FA > 0. On the right-hand side, all three fibers experience the same amount of tissue damage, such that all three eigenvalues (λ_1_, λ_2_, and λ_3_) are increased proportionally, resulting in a constant (unchanged) FA despite a net decrease in white matter fiber density. Because it is a relative measure, FA cannot provide quantitative information about net tissue differences/changes in the presence of crossing fibers. Bold arrow represents a relatively large increase (λ_1_ in part **A**), thin arrows represent a relatively small increase (λ_1_ and λ_2_ in part **A**), and medium arrows indicate a moderate increase (λ_1_, λ_2_ and λ_3_ in part **B**).

Based on the conventional interpretation, one might erroneously infer that a higher FA value reflects an increased number of microstructural tissue elements within the underlying white matter (e.g., higher fiber density, lower membrane permeability, greater myelination, etc.). However, this is not necessarily the case. Due to the presence of crossing fibers throughout the majority of cerebral white matter, disproportionate atrophy or degradation of one or more fiber bundles – along with the relative preservation of other fiber bundle(s) – could result in a seemingly paradoxical increase in FA, despite an actual decrease in local fiber density, myelination, etc., (Figure 2A). In fact, increased diffusion anisotropy in the presence of Wallerian degeneration due to chronic lacunar infarcts has been reported (Pierpaoli et al., 2001), where degeneration of motor pathways in the rostral pons caused the transverse pontine fibers to become the dominant pathway – ultimately changing both the FA value as well as the principle diffusion direction. It should be noted, however, that if the Wallerian degeneration had instead targeted the pontine fibers, the motor pathways could have remained unchanged, with FA still showing an increase and relatively little effect on the principle diffusion direction.

Although it is perhaps self-evident, we would briefly point out that the opposite effect can also be true – where FA values could decrease due to a disproportionate increase in one or more of the non-dominant fiber bundles (e.g., during neurodevelopment, neuroplasticity and/or neural repair). For example, if the motor pathways remain constant while the transverse pontine fibers mature (or undergo neural repair, based on the example above), one might observe lower FA values in the rostral pons, despite a net increase in local white matter fiber density.

### Thought Experiment #2: Interpreting Fractional Anisotropy Equivalence or Stability

Based on the conventional interpretation, one might erroneously infer that equal FA values reflect equivalent underlying tissue composition. However, as in the example above, this is not necessarily the case in the presence of crossing fibers either. Indeed, anytime there is a proportional change in the three eigenvalues (λ_1_, λ_2_, and λ_3_), FA will remain constant. Therefore, in the case of neurodevelopment, aging, traumatic brain injury, and/or neurologic disease, real differences could be missed if the underlying fiber bundles in a given region differ in the same way or change at the same rate, relative to each other. For example, if a multiple sclerosis (MS) lesion or traumatic brain injury damages all of the underlying fiber bundles within a particular region equally (e.g., increased membrane permeability and decreased myelination leading to an equal reduction in λ_1_, λ_2_, and λ_3_), FA will appear unchanged despite potentially significant alterations in the underlying tissues (Figure 2B). Of course, the opposite effect could also be true during neurodevelopment, where FA could appear constant if the all of the constituent fiber bundles were to mature at the same rate (i.e., equal increase in λ_1_, λ_2_, and λ_3_).

With that being said, it is perhaps important to briefly comment on the difference between forward and reverse inferences with respect to FA stability. For example, the aforementioned intra-scanner and inter-scanner repeatability studies acquired test-retest scans over a short interval from the same healthy control participants (Vollmar et al., 2010; Luque Laguna et al., 2020). Given the tightly controlled nature of these experiments, no underlying tissue changes were anticipated, so it was reasonable to expect stable FA values (i.e., forward inference). However, this is not the same as trying to infer a lack of tissue changes from stable FA values under less well-controlled experimental conditions (i.e., reverse inference).

## General Discussion

These examples hopefully highlight how biophysical interpretations of FA, AD, and RD values are problematic in the presence of white matter fiber crossings, and how interpreting them to reflect similarity, stability, differences or changes in tissue microstructure can potentially lead to Type I (false positive) and/or Type II (false negative) errors. It is perhaps also worth noting that *ex vivo* MRI and histological studies of the macaque brain have revealed that white matter crossing fibers become even more apparent upon higher resolution and more detailed examination, suggesting that this is a fundamental problem that cannot be overcome by scanning at higher resolution to reduce partial volume effects (Schilling et al., 2017). Therefore, there is no way of controlling for these effects or estimating the prevalence of Type I and Type II errors (which could even occur simultaneously in different brain regions) inherent in any reverse inferences between FA, AD or RD and the underlying tissue microstructure.

For simplicity, we have focused on FA in our examples, but it should be noted that AD and RD are confounded for similar reasons. It was pointed out using simulations and a review of empirical investigations more than a decade ago that changes in AD can induce spurious alterations in RD and vice versa in voxels containing crossing fibers (Wheeler-Kingshott and Cercignani, 2009), which we now know account for the majority of cerebral white matter regions.

This does not, however, mean that all of the previous interpretations using these metrics are necessarily wrong – especially in cases where the findings have been corroborated using other imaging metrics. For example, there are obviously certain white matter regions with very few crossing fibers (e.g., corpus callosum, corticospinal tract, spinal cord, etc.), where the interpretation of these metrics is more straightforward. Moreover, as pointed out by Jeurissen et al. (2013), even regions with crossing fibers, many observed differences/changes in FA, AD and/or RD are likely to reflect actual anatomical and biophysical phenomena, as long as other pitfalls in the acquisition and analysis have been avoided (Jones and Cercignani, 2010). However, it suggests that strong biophysical interpretations and conclusions should not be drawn using FA, AD or RD measurements alone, and that these should be complimented by other brain imaging metrics that are more robust to the presence of multiple fiber populations and complex fiber geometries.

## Potential Alternatives to Fractional Anisotropy, Axial Diffusivity, and Radial Diffusivity

All MRI methods have limitations that researchers need to consider when drawing conclusions. Therefore, with the caveat that each of the following methods has their own limitations, we would like to briefly present a few alternatives (albeit not a comprehensive list) of alternative quantitative MRI methods that are likely to be more robust to the presence of white matter crossing fibers.

Staying within the DTI framework, we would suggest that either the MD (i.e., mean apparent diffusion along the three tensor dimensions; MD = [λ_1_ + λ_2_ + λ_3_]/3) or the Trace (i.e., total apparent diffusion along the three tensor dimensions; Trace = [λ_1_ + λ_2_ + λ_3_]) values are likely the most robust and interpretable metrics for drawing reverse inferences about the underlying tissue characteristics. Unlike FA, these are not proportional/relative metrics; and unlike FA, AD, and RD, they not only account for, but equally weight, the amount of diffusion along all three axes of the diffusion tensor. As a result, MD and Trace values are theoretically more robust to multiple fiber populations and/or complex fiber geometries, and are likely the best DTI indicators of how tissues are constraining diffusion within a voxel. In both of the theoretical thought experiments outlined in Figure 2, MD and Trace values would increase as expected (owing to less restricted diffusion within the voxel). This is also supported by empirical findings that MD is more closely related to neurite density than FA, AD or RD measures (Genc et al., 2017).

Within dMRI, but including more advanced diffusion analysis approaches, there are several models that are more robust to crossing fibers. These include, but are not limited to: diffusion kurtosis imaging (DKI) (Jensen et al., 2005; Jensen and Helpern, 2010), neurite orientation dispersion and density imaging (NODDI) (Zhang et al., 2012), tensor-valued diffusion encoding or b-tensor encoding (Szczepankiewicz et al., 2016, 2019), and novel fixel-based analysis approaches (Raffelt et al., 2017). For example, DKI data acquired using conventional hardware and reasonable scan times can resolve crossing fibers significantly better than conventional DTI (Glenn et al., 2016), and certain DKI metrics such as kurtosis fractional anisotropy (KFA) and quantitative kurtosis tensor measures (e.g., radial tensor kurtosis) are likely more robust to crossing fibers (Hansen et al., 2016; Hansen and Jespersen, 2016). Alternatively, NODDI can estimate neurite density in dendrites and axons, as well as orientation dispersion (i.e., fanning of neurites), which are factors that contribute to, but are distinct from DTI-based FA measures. Orientation dispersion estimates the angular variability between neurites and provides a better measure than FA in regions with fanning or crossing fibers. Moreover, tensor-valued diffusion encoding allows the estimation of a microscopic fractional anisotropy (μFA), which disentangles orientation dispersion from microscopic anisotropy at a sub-voxel level and could overcome the problem of crossing fibers (Szczepankiewicz et al., 2015). Finally, recent fixel-based analysis approaches have been proposed to characterize a specific fiber population within a voxel (i.e., a “fixel”) (Raffelt et al., 2017). Modeling individual fibers at the sub-voxel level like this could lead to more sensitive measurements and more detailed understandings about tissue degeneration in various disorders (Finkelstein et al., 2021).

Beyond dMRI approaches, other quantitative MRI techniques such as calibrated T1w/T2w ratio mapping (Ganzetti et al., 2014; Uddin et al., 2018), inhomogeneous magnetization transfer (ihMT) imaging (Manning et al., 2017; Swanson et al., 2017), and T2 relaxation-based myelin water imaging MWI (Prasloski et al., 2012; Lee et al., 2018) are likely less affected by fiber orientation; and in the case of ihMT and MWI likely also provide more myelin-specific information than can be obtained with more general measures based on DTI or T1w/T2w ratio metrics (Mädler et al., 2008; Ercan et al., 2018; Uddin et al., 2019).

## Conclusion

We now know that the majority of cerebral white matter voxels contain multiple fiber populations and complex fiber geometries, and that increases, decreases, and indeed even stable FA, AD, and RD measures become difficult (if not impossible) to interpret in terms of the other underlying tissue microstructural properties (e.g., fiber diameter, fiber density, membrane permeability, myelination, etc.). On the contrary, given that these measures are highly sensitive to the relative volume fractions of the various fiber populations, the microstructural integrity of each constituent fiber population, and any combination of changes between these factors, extreme care should be taken when drawing conclusions about the biophysical underpinnings of FA, AD, and RD values. Except for specific regions (e.g., corpus callosum, spinal cord, etc.) where complex fiber geometries are not generally be expected, we would encourage future studies to use other MRI metrics that are more robust to the presence of crossing fibers – either instead of, or (at minimum) in addition to DTI-based FA, AD, and/or RD values.

## Author Contributions

CRF drafted the initial manuscript. However, all authors contributed to the manuscript conception, literature review, approved the final version prior to publication, and revised it critically for important intellectual content.

## Conflict of Interest

The authors declare that the research was conducted in the absence of any commercial or financial relationships that could be construed as a potential conflict of interest.

## Publisher’s Note

All claims expressed in this article are solely those of the authors and do not necessarily represent those of their affiliated organizations, or those of the publisher, the editors and the reviewers. Any product that may be evaluated in this article, or claim that may be made by its manufacturer, is not guaranteed or endorsed by the publisher.

## Footnotes

1 Since the intention of this paper is to guide the design and interpretation of future studies, rather than to point out mistakes or limitations in previous work, we will not pick on any specific papers that have used FA, AD, or RD measures without considering these limitations. However, since the interpretation of these metrics in this way continues to be a common practice, we feel that it is necessary to draw broader attention to this issue.
